# Host factors facilitating SARS‐CoV‐2 virus infection and replication in the lungs

**DOI:** 10.1007/s00018-021-03889-5

**Published:** 2021-07-05

**Authors:** Sébastien Boutin, Dagmar Hildebrand, Steeve Boulant, Michael Kreuter, Jule Rüter, Srinivas Reddy Pallerla, Thirumalaisamy P. Velavan, Dennis Nurjadi

**Affiliations:** 1 Department of Infectious Diseases, Medical Microbiology and Hygiene, University Hospital Heidelberg, Im Neuenheimer Feld 324, 69120 Heidelberg, Germany; 2 Translational Lung Research Center Heidelberg (TLRC), German Center for Lung Research (DZL), University of Heidelberg, Heidelberg, Germany; 3 Division of Cellular Polarity and Viral Infection, German Cancer Research Center (DKFZ), Heidelberg, Germany; 4 Department of Infectious Diseases, Virology, University Hospital Heidelberg, Heidelberg, Germany; 5 Center for Interstitial and Rare Lung Diseases, Pneumology, Thoraxklinik, University of Heidelberg, Heidelberg, Germany; 6 Institute of Tropical Medicine, Universitätsklinikum Tübingen, Tübingen, Germany; 7 Vietnamese-German Center for Medical Research, Hanoi, Vietnam

**Keywords:** SARS-CoV-2, COVID-19, Innate immune response, Co-morbidities, Host factor, Microbiome

## Abstract

SARS-CoV-2 is the virus causing the major pandemic facing the world today. Although, SARS-CoV-2 primarily causes lung infection, a variety of symptoms have proven a systemic impact on the body. SARS-CoV-2 has spread in the community quickly infecting humans from all age, ethnicities and gender. However, fatal outcomes have been linked to specific host factors and co-morbidities such as age, hypertension, immuno-deficiencies, chronic lung diseases or metabolic disorders. A major shift in the microbiome of patients suffering of the coronavirus disease 2019 (COVID-19) have also been observed and is linked to a worst outcome of the disease. As many co-morbidities are already known to be associated with a dysbiosis of the microbiome such as hypertension, diabetes and metabolic disorders. Host factors and microbiome changes are believed to be involved as a network in the acquisition of the infection and the development of the diseases. We will review in detail in this manuscript, the immune response toward SARS-CoV-2 infection as well as the host factors involved in the facilitation and worsening of the infection. We will also address the impact of COVID-19 on the host’s microbiome and secondary infection which also worsen the disease.

## Introduction

In December 2019, a new coronavirus named as the severe acute respiratory syndrome coronavirus-2 (SARS-CoV-2 or 2019-nCoV) was found responsible for acute atypical respiratory disease. This virus shared a strong homology with SARS-CoV, which was responsible for acute respiratory distress syndrome (ARDS) and high mortality in 2002–2003 [1]. SARS-CoV-2 (Severe Acute Respiratory Syndrome Corona Virus 2) is the etiological agent of Coronavirus disease 2019 (COVID-19) [2–4]. The first cases were reported at the end of 2019 in Wuhan, China. The virus quickly spread globally, and as of early fall 2020, more than 30,000,000 people have been infected by SARS-CoV-2 worldwide. SARS-CoV-2 is infecting primarily the respiratory system and caused the following symptoms: fever, dry cough and dyspnea [5] as well as lung abnormalities such as reduced lung function and pulmonary fibrosis [6]. Additionally, other organs can be affected, causing headache, dizziness, generalized weakness, digestive symptoms, vomiting and diarrhea [7, 8]. This broad range of symptoms is also heterogenous in the infected population with asymptomatic individuals and patients reaching significant hypoxia and in some cases ARDS. Severe disease progression often leads to a fatal outcome, with an increased incidence of mortality in the older population [9–11].

The management of the disease is limited to symptomatic treatment to decrease the severity of the symptoms as no curative therapy is available. To limit the spread of the virus, many countries decide to implement social distancing and lockdown. Many drugs such as antiviral agents, treatment to treat super-infection such as empirical antibiotics and antifungal drugs, have been tested in clinical trials without reaching a consensus on a definite therapy [12–14]. The course for a vaccine has also been initiated and became a worldwide priority leading to several candidate vaccines are now in clinical phase 3 [15–18].

**Fig. 1:**
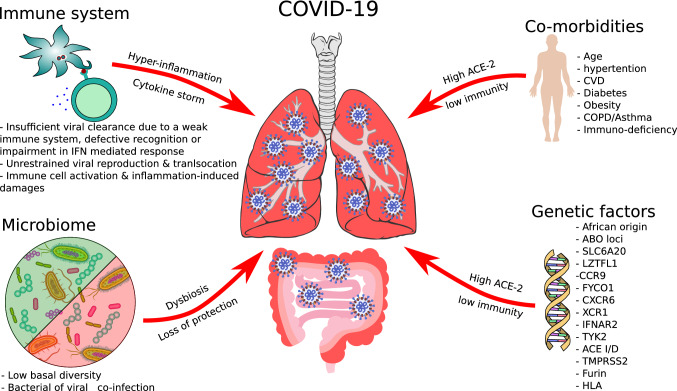
Impact of immune system, co-morbidities, genetics and microbiome as host factors on the infection by SARS-CoV-2 and the progress of COVID-19

However, the gain of knowledge of the host factor influencing SARS-CoV-2 virus infection and replication in the lungs is crucial for the development of an appropriate therapeutic approach. We will review the clinical aspects and basic features of SARS-CoV-2 and its impact of immune response and discuss the influence of co-morbidities, co-infection and microbiome changes on the course of the disease (Fig. 1).

## Etiology of SARS-CoV-2

The precise origin of SARS-CoV-2 is still under debate; however, it is now broadly accepted that this virus, like its predecessors SARS-CoV and MERS-CoV (Middle East respiratory syndrome), has a zoonotic origin. It is still unclear whether SARS-CoV-2 has jumped directly from the bat to humans or whether there was an intermediate host where the bat coronavirus has evolved to jump into humans (pangolin, snakes, turtles and feral dogs) [19]. SARS-CoV-2 belongs to the family *Coronaviridae* genus betacoronavirus. Based on phylogeny, the virus was classified in a sister clade to the prototype human and bat severe acute respiratory syndrome coronaviruses (SARS-CoVs) of the species severe acute respiratory syndrome-related coronavirus [20].

SARS-CoV-2 like all *Coronaviridae* are single-stranded positive-sense enveloped RNA viruses which carry the largest genomes (26–32 kb) among all RNA virus families. Transcription of the SARS-CoV-2 genome is very complex and leads to the production of one full-length genomic and nine subgenomic RNAs all of which contain a 5′-cap structure and a 3′ poly(A) tail. Additionally, it was reported that non-canonical subgenomic RNAs are also transcribed through fusions, deletions, and/or frameshifts, but their functions remain elusive [21].

### Infection by SARS-CoV-2

The life cycle of SARS-CoV-2 initiates with the spike proteins (S) located at the surface of the viral envelope binding the cellular receptor angiotensin-converting enzyme 2 (ACE2) [22]. Virus entry does not only rely on the cellular receptor ACE2 but also on the cellular serine proteases (TMPRSS2, TMPRSS11D and TMPRSS13), cysteine proteases, cathepsins B, L (CTSB/L) and furin that activate the spike protein through proteolytic cleavage [22–25]. Following entry and release into the cytosol, the full-length genomic mRNA is translated into 16 non-structural proteins from two distinct open reading frames (ORFs). The viral genome is then used both as a template for the transcription of the different subgenomic RNAs and as a template for replication by the RNA-dependent RNA polymerase nsp12 [21]. Viral replication takes place in virus-induced replication organelles that are derived from intracellular membranes. These replication organelles are formed by endoplasmic reticulum-derived convoluted membranes that form double-membrane vesicles and open double-membrane spherules [26, 27]. Viral synthesis happens in the double-membrane vesicles [28] and, by analogy to SARS-CoV and MERS-CoV are believed to be induced by the viral protein nsp3 and nsp4 [27]. Upon infection, infected cells can detect the presence of viruses using pathogen recognition receptors (PRRs) which sense molecular signatures induced or directly associated with the pathogen or pathogen-associated molecular patterns (PAMPs).

### Immune response to SARS-CoV-2

To date, how SARS-CoV-2 is sensed by infected cells and how the cells respond to viral infection is still under research. According to the composition of the RNA virus with different PAMPS and its replication cycle, sensing by extracellular as well as intracellular Toll-like receptors (TLRs) such as TLR3, TLR4 and TLR7/8 and retinoic acid-inducible gene-I-like receptors (RLRs) (RIG-I and MDA-5) seemed likely and has been shown meanwhile in different studies [29–31]. For example, it has been found that the spike protein of SARS-CoV-2 shows binding efficiency with the extracellular domains of TLRs including TLR1, TLR4 and, TLR6, with the strongest affinity with TLR4 [32]. Furthermore, studies on in‐silico multi‐epitope‐based peptide vaccine candidates against the SARS‐CoV‐2 has proven effectively binding with TLR3, TLR4 and, TLR5 and activating of subsequent signaling pathways and inflammation [33]. Additionally, mRNA of SARS-COV-2 NSP10, S2, and E proteins are considered as possible virus‐associated molecular patterns that bind to TLR3, TLR9, and TLR7, respectively [34]. At this, it has been shown that targeting human TLRs with TLR agonists and thereby inhibiting SARS‐CoV‐mediated TLR activation and expression of pro‐inflammatory cytokines might be therapeutically useful in COVID‐19 [35].

Interestingly, a recent study suggests that the cGAS–STING pathway might also be involved in sensing SARS-CoV-2 infection [36] and this observation is supported by the fact that polymorphisms in the STING pathway might be associated with the pathogenesis of COVID-19 [37]. Obviously, precondition of a protective antiviral immune response is the recognition of viral structures. Hence, genetically encoded defects regarding the viral recognition could worsen an infection. Indeed, Van der Made et al*.* could link loss-of-function mutations in the TLR 7 (sensor of viral RNA) [38] in myeloid cells, to a critical course of COVID-19 in otherwise healthy young men [39].

Recognition of PAMPs by PRRs induce a complex signaling cascade in the host cell. In consequence, a range of transcription factors are activated that drive the production of interferons (IFNs) and pro-inflammatory molecules [40, 41], indispensable for the full induction of the immune system. Especially, IFNs are considered as key antiviral cytokines in antiviral (including Coronaviruses) response as they induce the production of hundreds of interferon-stimulated genes (ISGs) known to exert broad antiviral functions [42–45]. In response to these cellular antiviral strategies, coronaviruses are developing a broad range of countermeasures to evade and interfere with the cell intrinsic innate immune response [29, 46]. SARS-CoV-2 has been shown to be able to interfere directly with the production of IFN and with the IFN-mediated production of ISGs [47, 48]. On the host side, impairment of IFN-mediated antiviral response is mediated by type I interferon neutralizing antibodies [49]. Bastard et al*.* report that 101 of 987 patients with life-threatening COVID-19 pneumonia had neutralizing IgG auto-Abs against type I IFNs, at the onset of critical disease and absent in 663 individuals with asymptomatic or mild SARS-CoV-2 infection. The overwhelming majority of antibody-positive patients were male [49]. In line with these observations, another study from Hadjadj et al*.* describes a distinct phenotype in severe and critical patients, consisting of a highly impaired type I interferon response, associated with a sustained blood viral load and a pronounced inflammatory response (TNF-α, IL-6) [50]. Accordingly, IFN neutralizing antibodies could inhibit elimination of the virus in the beginning of infection and thereby promote a severe and critical course. Importantly, neutralizing IgG auto-Abs against type I IFNs exist in almost all patients with autoimmune polyendocrinopathy syndrome type I (APS-1) [51] and are also found in women with systemic lupus erythematosus [52].

Virus-recognition and subsequent mediated inflammation and triggered adaptive immunity is crucial to fight and eliminate the pathogen. If the protective immune response is insufficient in the beginning, the virus can replicate with the consequence of virus-mediated impairments and host-mediated potentially life-threatening organ-restricted or systemic inflammation, culminating in a cytokine storm [53]. A cytokine storm can mediate various effects on the organism. Blood vessels are dilated, and blood pressure decreases, the epithelial barrier function is impaired, and the coagulase system is highly activated. As consequence, plasma leaks into the tissue, thrombi arise in the small vessels, and the oxygen supply of organ and tissue is insufficient. Organ failure and death are the most critical implications of a cytokine storm also observed in severe cases of SARS-CoV-2 infection [54]. The hyperinflammation in COVID-19 includes a variety of factors such as IFNs, chemokines, Colony-stimulating factors, TNF-α and interleukins (ILs), such as IL-6.

As ultimate result of the cytokine storm in SARS-CoV-2 infection, the ARDS might occur. ARDS is a hallmark of a critical course of COVID-19 and accounts for a significant number of deaths in patients [54]. ARDS is a form of severe hypoxemic respiratory failure characterized by inflammatory injury to the alveolar capillary barrier with extravasation of protein-rich edema fluid into the airspace. ARDS can be caused by bacterial sepsis as well as SARS-CoV and MERS-CoV infections [55]. Kox and Kan with colleagues reported that cytokine levels in COVID-19 patients with developed ARDS are lower than in bacterial sepsis, the most common risk factor for ARDS [56]. However, the threshold are still sufficient to induce inflammation-mediated pathomechanism [57, 58]. Evidence points towards a key role of thromboembolism and hypercoagulability that sustain pro-inflammatory cytokines-mediated effects leading to multiorgan failure [59].

Obviously, the immune response codetermines the outcome of SARS-CoV-2 infection. Predisposing host factors that might favor a severe COVID-19 course include those who inhibit the protective immune response on the first side and/or sustain a hyperinflammation at later stages of infection. On the cellular level, several immune cells are involved. Important players in the antiviral response are natural killer (NK) cells. NK cells are innate lymphocytes that circulate in the blood and infiltrate in parenchyma of peripheral tissue such as the lung upon infection [60]. The activation status of NK cells is regulated by the outcome of several activating and inactivating receptors-mediated signaling. Major histocompatibility complex (MHC) class I molecules are ligands for inhibitory or activating receptors [61]. Engagement of these receptors modulates NK cell responses and T-cell antigen receptor (TCR)-dependent T-cell activation. Expression of MHC class I is frequently impaired in virus-infected cells, which results in abolishment of NK cell inhibition and induction of the effector functions [61]. Full activation of NK cells requires type I IFNs or pro-inflammatory cytokines (such as IL-15, IL-12, IL-18) [62] released by infected and activated immune and non-immune cells, such as airway epithelial cells [63, 64]. Once activated NK cells can, depending on the subtype, kill target cells and release inflammatory cytokines and chemokines, thus participating in the recruitment and activation of other leukocytes [65]. Given the importance of NK cell activity in early viral clearance and late immunopathology, a direct role for NK cells is suggested. However, the role of NK cells in COVID-19 severity has just begun to be analyzed. So far generated data report alterations in the NK cell numbers, function and phenotype that are associated with COVID-19 severity. A study from Hadjadj et al*.* using peripheral blood from COVID-19 patients shows that NK cell numbers inversely correlated with disease severity. Here, infiltration of NK cells into the lung cannot be excluded. Furthermore, cells in patients with active disease highly expressed the inhibitory receptor NKG2 and displayed a hyporesponsive phenotype in respect of production of IFN-γ, TNF-α, IL-2 and granzyme B [66]. Another study from Liao et al*.* could show that upon infection with SARS-CoV-2 NK cell infiltration of the lungs increases. Nevertheless, in severe disease, the proportions of NK cells were reduced with a high expression of inhibitory receptors [67]. A similar result was postulated by Carvelli and colleagues in peripheral blood. Absolute numbers of NK cells were significantly reduced in COVID-19 patients with developed pneumonia and ARDS compared to healthy controls and inhibitory receptors were more abundant on the cells [68]. Wilk et al*.* report from their study that CD56bright (cytokine producers) population was depleted in peripheral blood of all COVID-19 patients but the CD56dim (cytotoxic) population was depleted only in patients with severe COVID-19 [69]. Finally, Maucourant et al*.* claim that severe hyperinflammation is associated with the proliferation and activation of ‘adaptive’ NK cells, a sub-population with enhanced antibody-dependent cellular cytotoxicity, as well as the arming of CD56bright NK cells with cytotoxic molecules. These results suggest that a distinct NK cell immunophenotype is associated with the severity of COVID-19 [70]. However, further studies need to be done to gain a clearer picture about the role of NK cells in disease morbidity.

Naturally, besides NK cells further cells of the innate arm of the immune system are modulated in SARS-CoV-2 infection and contribute to the clearance of infection as well as the course of disease. For the highly plastic monocyte population, it was shown that patients with mild COVID-19 symptoms have high levels of inflammatory monocytes, which most likely contribute to an effective fight against the virus. In contrast, monocytes in more severe COVID-19 courses show an anti-inflammatory functional phenotype [71]. This points towards a reprogramming of monocytes during infection from an immunogenic to an immunosuppressive character that is also observed in bacterial infection and sepsis [72, 73] and could be critical in terms of complete clearance of SARS-CoV-2 and possible secondary infections. Not surprisingly, also the granulocyte population is altered in COVID-19. Neutrophils are the most common white blood cells and one of the first cells to migrate to the site of infection. Interestingly, abundance of immature neutrophil precursors is increased in patients with mild and severe courses. Additionally, Schulte-Schrepping et al. observed a correlation between disease severity and this emergency myelopoiesis [71]. In general, in SARS-CoV-2 infection, resting neutrophils seem to be replaced by various neutrophil clusters, including inflammatory, immunosuppressive as well as immature cells [74]. In more severe cases, mature neutrophils were shown to possess a rather immunosuppressive then immunogenic phenotype depicted by the expression of PD-L1 and impaired oxidative burst response [71]. This supports the finding that critical ill COVID-19 patients eventually fall into a state of immune silence after a period of excessive inflammation [74].

Importantly, lymphocytes of the adaptive arm of the immune system, seems to be important for COVID-19 outcome. In general, T cells are indispensable for coordination of antiviral immune responses. Activated by antigen-presenting cells, they regulate innate immunity, activate the humoral response, limit viral replication and remove infected cells [75]. Hence, T cells promote protective immunity against SARS-CoV-2 but also might sustain pathogenesis of COVID-19 when an exaggerated or misdirected T-cell response takes place. However, the impact of different subsets of T cells in protection or pathogenesis and severity is not solved yet. To put it simply, CD4+ T cells help B cells to produce antibodies and macrophages to phagocytose and CD8+ T cells kill virus-infected cells [76]. Hence, both subtypes should limit viral replication. By consensus SARS-CoV-2 infected persons develop a strong and broad (not specific for spike2) CD4 T-cell as well as CD8 T-cell response including the development of memory lymphocytes, suggesting a long-term immunity [77]. Indeed, long-term T-cell-mediated immunity gained during infection with another coronavirus are accused to ensure a prompt and protective response and a mild COVID-19 course [78, 79]. Furthermore, lymphopenia of T cells (and B cells) is a hallmark of COVID-19, is more pronounced in severely ill patients [80, 81] and might affect especially CD8 T cells [82]. In mild courses, more CD8+ T cells are found than in severe COVID-19 cases [83, 84]. In severe COVID-19 cases the T-cell response is dominated by spike-specific CD4+ T cells, and as consequence of T-cell and B-cell collaboration high numbers of neutralizing high-affinity-antibodies are detected [85]. As many studies have shown a higher viral load in more severe COVID-19 cases, the increase in CD4+ T cells in those with a severe outcome, might be only a result of the increased antigenic burden. Nevertheless, CD4+ T-cell and/or antibody responses could contribute to disease severity, rather than just reflecting it or contributing to a better viral clearance [85]. Further studies are needed to revise whether the early T-cell response is predictive of disease outcome.

### Comorbidities and risk factor in COVID-19

SARS-CoV-2 is capable of infecting people of all ages, but older people or people with pre-existing medical conditions showed a predisposition to infection and severe forms of COVID-19 [86]. The sensibility of the elderly could actually be explained by the over-expression of ACE2 in the elderly or the high prevalence of co-morbidities in this cohort [87]. The list of co-morbidities includes obesity, diabetes, hypertension, lung, liver, and kidney disease; immune-compromised patients (cancer patients on chemotherapy, transplant recipients), smokers and patients taking steroids chronically [88]. The order of importance varied between studies but the major factors seems to be hypertension and cardiovascular problem followed by diabetes and respiratory underlying diseases [5, 88–94].

Mechanistically, the risk factor hypertension for a severe COVID-19 course, could be linked to the use of angiotensin-converting enzyme (ACE) inhibitors and angiotensin receptor blockers (ARBs) to treat patients with hypertension. The manipulation of the renin–angiotensin system (RAS) could have an implication on the invasion and replication of SARS-CoV-2 in the cells. Those treatments are known to potentially increase the levels of ACE2 in several tissues which could increase the power of invasion by SARS-CoV-2 [95–97]. Studies have shown different results regarding the impact of such inhibitors with deleterious effect as demonstrated in patients with underlying cardiovascular disease [98] or a beneficial effect on the clinical outcomes of COVID-19 patients with hypertension [99]. Altogether, the lack of knowledge does not allow us to draw a causal conclusion on the risk factor associated with hypertension nor decide to stop the use of ACE inhibitors and ARBs [100].

An underlying cardiovascular disease (CVD) is linked to a worst outcome in patient with COVID-19. Patients with CVD develop severe form of COVID-19 and face more likely fatal outcome [101, 102]. The risk factors due to cardiovascular disease might depend on the same mechanistic as the hypertension because of the use of ACE inhibitors and ARBs. Furthermore, cardiac damage might occur during infection and treatment. Many antiviral drugs have an impact on cardiac insufficiency, arrhythmia or other cardiovascular disorders [103]. The virus itself, SARS-CoV-2 can induce myocardial injury, which is consistent with an increase in high-sensitivity cardiac troponin I (hs-cTnI) levels [5, 104]. Patients with COVID-19 often present heart palpitations and chest tightness and heart damage can occur even in the absence of underlying cardiovascular damage. The mechanism is still unclear, but the over-response of the cytokine storm syndrome might be the cause of the damage to the myocardial cells [105]. Furthermore, SARS-CoV-2 infection can also affect the heart of patients who have recovered from the infection, indicating a long-lasting impact on the cardiac system. Many patients showed post-COVID-19 myocardial dysfunction independently of the pre-existing cardiovascular disease [106, 107].

Metabolic disorders (MDs), such as diabetes and obesity are associated with a pro-inflammatory, and prothrombotic state that can promote atherosclerosis (accumulation of lipids and leucocytes in blood vessels, leading to the formation of plaque) that restricts blood flow and promotes organ dysfunction due to insufficient oxygen supply [108]. Hence, MDs are considered as major risk factor for a critical course of COVID-19 [109]. Diabetes, a common comorbidity in patients with COVID-19, is a multifaceted metabolic disorder affecting the glucose level in the organism. As a result of an absolute or relative insulin deficiency or resistance to its action, impaired glucose tolerance and hyperglycaemia takes place [110]. A small part of patients treated for diabetes also receive ACE inhibitors and ARBs, which could impact the prevalence of infection in this cohort [111]. In fact, diabetes is associated with a poorer outcome in COVID-19. This is most probably due to chronic inflammation with promoted atherosclerosis that sustains the symptoms in severe COVID-19 [112]. However, the underlying reasons for predisposing morbidity are complex. In addition to the general impairments in immunity (impaired neutrophil chemotaxis, phagocytosis, T-cell function) by which diabetes predisposes to infectious disease, diabetes might facilitate virus entry and replication. Roca-Ho et al*.* could show that diabetic mice have increased expression of ACE-2 in renal cortex, liver and pancreas. Although the upregulation of the receptor could not be confirmed in lungs this might predispose people with diabetes to infection with SARS-CoV-2 [113]. Furthermore, diabetes is associated with an increase in type-1 membrane-bound protease furin. Furin is involved in the entry of coronaviruses into the cell and therefore might facilitate viral entry and replication [114–116].

Obesity is defined by an excess accumulation of adipose tissue to an extent that impairs both physical and psychosocial health and well-being which can affect both children and adults [117]. Obesity is also linked to ACE2, adipose tissue express higher levels of ACE2 which might enhance the viral entry in adipocytes [118, 119]. Therefore, a concern was raised if adipose tissue can serve as a reservoir for the spread of SARS-CoV-2 [120]. Furthermore, obesity does not come alone and is often associated with other co-morbidities which are also increasing the risk of COVID-19 such as hypertension, diabetes, cardiovascular diseases and lung function decline. It is closely associated with the development and worsening of type 2 diabetes [121], implicating similar reasons for the reported worsening COVID-19 outcome like diabetes [122]. Briefly, the major reasons for promoting morbidity most probably include chronic inflammation and facilitated thrombosis. Dead and dying adipocytes, mediate infiltration and activation of macrophages, that release high levels of cytokines such as TNF-α, IL-6, and IL-1β [123]. This increased inflammation might contribute to alveolar damage or systemic organic dysfunction in critical cases. Additionally, obesity-associated thrombosis [124] obviously displays the metabolic disease as a risk factor for severity in COVID-19 that can be viewed as prothrombotic disease and treated in some cases with heparin beneficially [125]. Furthermore, released hormones could be important. Obesity is associated with higher circulating leptin and lower circulating adiponectin. Adiponectin is considered as anti-inflammatory, and adiponectin-deficient mice develop inflammation of the pulmonary vasculature [126, 127]. This might account for facilitating a severe COVID-19 course [128]. However, some risk groups tend to have lower and some tend to have higher adiponectin levels [129, 130] emphasizing the importance of the sum of factors for disease outcome. In summary, the factors displaying obesity as a risk factor in SARS-CoV-2 infection is complex and additional factors that might contribute are nicely reviewed by Lockhart and Stephen [122].

Within the high range of chronic respiratory distress, chronic obstructive pulmonary disease (COPD) and asthma were the most associated as comorbidity [5, 92]. COPD and asthma were already associated with other coronavirus infection, such as SARS-CoV and MERS-CoV [131]. However, in case of COVID-19, data are not clear if COPD really increases the rate of infection, but it is clear that patients with COPD are most likely to present severe symptoms [132, 133]. Most COPD patients have a long history of smoking or exposure to other harmful particles or gases, capable of impairing pulmonary defences even years after the absence of exposure [134, 135]. COPD is characterized by persistent respiratory symptoms and airflow limitation due to airway inflammation and/or alveolar abnormalities [136]. COPD patients showed an odds ratio of 2.681 for ICU admission, mechanical ventilation or death, even after adjustment for age and smoking [137]. They also are more prevalent in severe cases and dying cohort [9, 104, 137–139]. The mechanism favoring severe infection and worse outcomes for COPD patients is unclear but elevated ACE2 expression is associated with COPD and smoking [140, 141]. Leung and colleagues demonstrated in three separate COVID-19 cohorts with available gene expression profiles from bronchial epithelial cells that ACE-2 expression was significantly elevated in COPD patients compared to control subjects [140]. This might facilitate replication of the virus. Furthermore, impairment in the protective immune response in the beginning of infection is also probable as innate as well as adaptive immunity towards pathogens is impaired in COPD [135, 142]. For example, the number of mature and thereby T-cell activating dendritic cells is rather low in COPD patients [143]. The numbers of alveolar macrophages are increased in COPD patients. However, their effector functions such as phagocytosis of pathogens, production of pro-inflammatory cytokines and antimicrobial factors, are downregulated [144]. Importantly, type I IFN production in lung epithelium and alveolar macrophages in response to viral infection is impaired in COPD [145]. Furthermore, COPD patients are less capable in producing mucosal virus-neutralizing sIgA [146]. Finally, also T-cell subsets are strongly affected in COPD. For example, CD8 T cells display impaired cytotoxic activity and an upregulation of inhibitory receptor programmed cell death protein-1 (PD-1). Importantly, COPD patients exhibit decreased numbers of pulmonary Treg cells, as well as reduced levels of FoxP3 mRNA and lung interleukin 10 secretion [147]. As regulatory T cells (Tregs) are essential to keep immune response and inflammatory reaction under control this in addition to increased ACE receptor and impairments in protective immunity, strongly suggests a predisposition of COPD patients for a critical COVID-19 course. Asthma is already known to lead to a higher sensitivity to develop viral infections due to a delayed innate antiviral immune response and impaired secretion of IFN-λ [148]. However, there is a clear lack of evidence in the case of COVID-19 despite the claim that asthma is one of the comorbidity in some cohorts [92]. The rate of asthmatics infected seems low compare to the normal population rate in some other country and their odds ratio toward severity was not increased [149, 150]. This would go in line with a study comparing COPD and asthma, showing that asthmatic patients in contrast showed a lower risk of severe outcome compared to COPD [151]. The authors also showed that the ACE2 expression and protein level was significantly decreased in asthmatics compared to healthy controls and COPD patients. The most probable explanation in the heterogenous findings in literature is that asthma is often associated with other complications such as obesity [152, 153]. With regards to other chronic lung diseases, interstitial lung diseases (ILD), data are sparser. Recently, it was shown that in patients with pre-existing ILDs hospitalization for COVID-19 is associated with a higher mortality, especially in those with idiopathic pulmonary fibrosis and those with a more advanced disease [94].

## COVID 19: relevance of host genetic factors

While age and co-morbidities largely determine the clinical course of COVID-19, equally host genetic predisposition for life-threatening COVID-19 is being increasingly recognized in world populations. Identification of such host genetic factors associated with different clinical phenotypes help disentangle elements associated with susceptibility, hospitalization, virulence.

Clinical outcomes of COVID-19 have been shown to be associated with interindividual, as well as interpopulation, differences. While some African nations and South Asian countries with young populations report low incidence and mortality, genetic predisposition may limit viral infection and modulate COVID-19 immunopathogenesis [154]. For instance, irrespective of age, sex and co-morbidities, individuals with African descent in the UK and United States are associated with susceptibility [155], hospitalization [156, 157] and mortality [156]. Similar observations have been reported in non-Hispanic black, Hispanic, and non-Hispanic American Indian or Alaskan Native populations [158] and among few Asian population with hospitalization and mortality [155].

Both monogenic and polygenic risk variants can predispose to disease progression. In COVID-19, it is polygenic risk variants, as multiple loci are thought to influence the clinical phenotype [159]. Various genome wide association studies (GWAS) and candidate gene studies have investigated the association of inter- and intra-ethnic genetic variations with COVID-19 clinical phenotypes. Among the well-studied loci of interest are blood group ABO, genes that facilitate SARS-CoV-2 entry into epithelial cells, and genes that modulate innate and adaptive immune responses.

In particular, the SNP rs11385942 on chromosome 3, located at 3p21.31 and surrounding the genes SLC6A20, LZTFL1, CCR9, FYCO1, CXCR6, and XCR1, was identified as a risk variant in three independent GWASs [160–162]. Although there is supporting evidence showing a significant association of SLC6A20 [163, 164], CCR9 [164], CXCR6 [165], and FYCO1 [166] with COVID-19 susceptibility, it is yet unclear on the functional role of these studied variants. In another GWAS study from the UK, the rs13050728 variant of interferon-alpha/beta receptor subunit 2 (IFNAR2), which binds to type 1 interferons and is known to modulate signal transduction, is associated with critical clinical outcome, alongside the rs11085727 variant in tyrosine kinase 2 (TYK2), which is implemental in cytokine signaling and interferon responses [161].

There is growing evidence from small observational studies to GWAS and country-level meta-regression analyses that the ABO blood group may play a role in the immunopathogenesis of SARS-CoV-2 infection, with group O individuals testing positive less frequently and group A having higher susceptibility to infection and a propensity for severe disease [167, 168]. The rs657152 at locus 9q34.2, was identified from two GWAS studies [160, 162]. Several hypotheses are postulated to explain the differences in SARS-CoV-2 infection by ABO type. The ABO blood group frequencies vary among human populations and the selective advantage of certain blood groups, perhaps related to exposure to specific pathogens, may have contributed to these variations. Also postulated are mechanisms that link blood type to individual risk for COVID-19: anti-A IgG antibodies in serum [169], lower ACE1 activity in blood type O [170], or the association of the ABO locus with different protein levels in plasma. These proteins include coagulation factors (factor VIII and von Willebrandt factor), IL-6 and TNF-α as inflammatory markers, and CD209 (DC-SIGN) [171]. CD209 is an L-type lectin expressed on dendritic cells and macrophages and has been proposed as an alternative entry receptor for SARS-CoV-2 [172], making elevated CD209 levels a likely risk factor for increased viral entry and more severe disease phenotypes.

SARS-CoV-2 infects lung alveolar epithelial cells using the primary entry receptor angiotensin-converting enzyme II (ACE2) [173]. High ACE2 expression in patients with co-morbidities are associated with severe COVID-19 [174]. It is not apparent whether high ACE2 expression is a risk for severe disease, as other entry receptors are increasingly recognized. Elevated angiotensin II levels have been shown to have pro-inflammatory and prothrombotic properties, resulting in poor clinical outcomes [175]. Equally, studies highlight the importance of a genetic deletion/insertion of a 285 bp Alu repeat sequence in intron 16 of the ACE gene fragment insertion (I allele) or the absence (D allele) [176]. ACE D allele was shown to increase expression of ACE2, and the D allele associated with COVID-19 severity [177] as well as frequency of hypoxia in SARS [178]. The ACE I/D allele distribution (rs4646994, ACE I/D polymorphism) from the Allele Frequency Database (ALFRED) (https://alfred.med.yale.edu/alfred/index.asp) shows that ACE D frequency is high in African ethnic groups [176]. Coding variants within the ACE2 gene have been investigated in genetic association studies, in vitro and in silico, but no risk variants have been determined to date [179]. Despite the entry receptor ACE2, cleavage of the spike protein is facilitated by several host proteases, including TMPRSS2 and furins. Latini et al. identified missense variants in furin and TMPRSS2 by whole-exome sequencing in hospitalized patients and observed significant differences in allele frequencies, particularly four variants that are common in patients (furin: rs769208985, rs114363287; and TMPRSS2: rs75603675, rs12329760) [180].

Human leukocyte antigens (HLA) are essential for antigen presentation, T-cell activation and adaptive immune response. In silico studies show that HLA-A*02:02, HLAB*15:03, HLA-C*12:03 exhibit increased binding affinity for SARS-CoV-2 peptides and thus are likely to elicit an effective adaptive immune response [181]. However, to date, only one GWAS in a Chinese cohort found a significant association for HLA types (HLA-A*11:01, B*51:01, and C*14:02) [182]. Furthermore, an Austrian study showed that the heterozygous HLA-E*0101/0103 variant and the HLA-E*0101 allele was associated more severe COVID-19 [183].

The molecular basis of viral inhibition is triggered by the first line of defense. The PRRs of innate immune system, such as TLRs recognizes the PAMPs activating the Interferon regulatory factors (IRFs) and the interferon related immune responses. TLRs are well studied as key transducers of host type I IFN responses during viral infections. SARS-CoV-2 is unique compared to SARS-CoV as it has been shown to evade the host interferon responses and replicate effectively in lung alveolar cells [184]. A strong host type I IFN responses during early stages of an infection are crucial in determining the COVID-19 clinical course. However, an imbalanced immune response characterized by poor production of type I IFN and increased release of pro-inflammatory cytokines contributes to the severe forms of the disease [31]. Nonsynonymous variants in type I IFN pathway [185] and autoantibodies against IFN1s [49] have been associated with low or undetectable type I IFN levels during SARS-CoV-2 infection and in patients with viral pneumonia.

Several host-derived factors may be useful as therapeutic targets. Compared to the beginnings of the COVID-19 pandemic, acceleration, and prioritization of research on SARS-CoV-2 infection have brought novel and insightful knowledge, which may be useful in understanding the mechanism of entry and in-host replication of the SARS-CoV-2 virus. While the expression of certain proteins, such as TMPRSS2, TMPRSS4, high-mobility group protein B1 (HMGB1) and cathepsin L, is associated with enhanced viral entry and replication, other proteins and genes, such as dipeptidyl peptidase 4 (DPP4), 25-hydrocholesterol (25HC), lymphocyte antigen 6E (LY6E), interferon-induced transmembrane proteins (IFITM), zinc finger antiviral protein (ZAP), heat shock protein 90 (HSP90), and apolipoprotein B mRNA editing enzyme catalytic polypeptide (APOBEC) has been identified as protective in the pathophysiology of SARS-CoV-2 infection. An overview of the function of a selection of these host-derived proteins in the context of SARS-CoV-2 infection is summarized in Table 1.

**Table 1:** Selected host-derived proteins with potential therapeutical use for SARS-CoV-2 infection

Protein	Name	Effect on COVID-19 disease progression	References
DPP4 (syn CD26)	Dipeptidyl peptidase 4	Direct involvement in COVID-19 disease progression not yet clear, but inhibition of DPP4 modulate inflammation and exert anti-fibrotic activity. High serum level may protect from infection through inhibition of viral binding to CD26	[321–324]
CH25H	Cholesterol 25-hydroxylase	Induced in COVID-19 patients. CH25H converts cholesterol to 25-hydrocholesterol (25HC). 25HC inhibits SAS-CoV-2 infection in lung epithelial cells and organoid models by blocking viral entry through depletion of membrane cholesterol	[325–327]
IFIH1 (syn. MDA5)	Interferon-induced helicase C domain-containing protein 1	Pattern recognition receptor, which can sense corona virus RNA (also known as MDA5). Low frequency allele SNP rs1990760 C > T is associated with lower IFN-b expression and increases susceptibility to SARS-CoV-2 infection	[328, 329]
IFITM, IFITM2, IFITM3	Interferon-induced transmembrane proteins (1–3)	Antiviral effector of antiviral activity of type I interferons against SARS-CoV-2 replication. Restriction of viral entry to low pH compartments. Inhibition of S-protein fusion	[330, 331]
LY6E	Lymphocyte antigen 6E	Restricts entry of corona viruses via interference of the S-protein fusion	[332, 333]
ZAP	Zinc finger antiviral protein	Expressed in human lung cells, endogenous ZAP expression reduces SARS-CoV-2 replication in human lung cells. Targets CpG dinucleotides of SARS-CoV-2	[334, 335]
HSP90	Heat shock protein 90	Inhibition of HSP90 activity can reduce viral replication and pro-inflammatory cytokine expression in airway epithelia	[336, 337]
APOBEC	Apolipoprotein B mRNA editing enzyme catalytic polypeptide	APOBEC protein family, together with tetherin and TRIM5a is part of the innate immunity against viral infections. Host-dependent genome editing of SARS-CoV-2	[338–340]

### Interaction between the host microbiome and viral infection

It is interesting to note that most of the comorbidity associated with COVID-19 such as hypertension, cardiovascular disease, diabetes or underlying respiratory disease is associated with a disturbance of the microbiome in gut or/and lung microbiome [186–191]. The microbiome is defined as the collection of genomes of all the microorganisms (bacteria, fungi, archaea and viruses) within a specific niche [192]. The human microbiome has such an impact in the health and functioning of our body that we are considered as a holobiont. The infection by SARS-CoV-2 begins in the lung and the major complications are lung infection and immune response dysregulation. Therefore, it has been hypothesized that the lung microbiome might be an important player in the initiation and progression of the disease [193]. In parallel, gut physiology seems to be affected during the disease as many patients suffer from diarrhea. This would also implicate a potential role of the gut microbiome, since the role of the gut microbiome in the gut physiology and the systemic immune response has been demonstrated [194, 195].

#### Lung microbiome

The lung has been for a long time considered a sterile organ, and every micro-organism found in the culture considered as pathogen [196]. However, nowadays, it is accepted that the lung is colonized by a diverse microbiome which contribute to build a proper immune homeostasis [197–201]. The constant exposure to microbes of the immune cells, primarily via γδ T cells, by microbes, will initiate the innate and adaptive immunity [202, 203]. The priming of immune cells by a rich airway microbiome avoid excessive immune response and a lower rate of allergic/asthmatic responses [189, 204, 205]. The protective role of the lung microbiome is due to the high diversity of organisms involved in both the immune priming and the competition within the microbiome. It has been documented in many studies that the occurrence of acute or chronic disease will modify the microbiome of the lung due to a disturbance of the balance colonization/elimination [206]. It has been demonstrated for many lung diseases such as ARDS, IPF, CF, COPD, bronchiectasis and asthma that the microbial structure of the lung is modified [207, 208]. Most of the time, the alpha diversity decreases with the establishment of a chronic infection leading to the dominance of single species. Most of the time, the dominant species belong to the phylum Proteobacteria that contains common lung-associated Gram-negative pathogens. Bacteroidetes and Firmicutes abundance decreases substantially during the establishment of the infection. However, it has not made clear yet if the alteration of the microbiome (dysbiosis) contributes to the disease progression or is a biomarker of the injury and inflammation.

The impact of viral infection on the lung microbiota is poorly studied. However, the accumulated knowledge on the upper airways gives us input to hypothesize that changes in the microbiome might follow viral infections and influence the severity of the disease via secondary infections [209]. Most of the studies on the impact of viral infections upon the microbiome have been focused on the upper respiratory tract due to the easiness to acquire samples. The diversity of the microbiome seems to be impacted by a viral infection, but results are variable depending on the viruses and the studies. An increase in the diversity following a viral infection has been correlated with patients infected with influenza [210] or H7N9 avian influenza [211]. On the other hand, a decrease in the alpha diversity was observed in patient with influenza but only in symptomatic patients [212] and in another study comparing several viral infections (influenza, parainfluenza, rhino, respiratory syncytial, corona, adeno, or metapneumo viruses) [213]. Interestingly, this study showed that the oropharyngeal microbiome was more correlated to the age than the type of viruses infecting the patients. The structure of the microbiome of the upper airways is also impacted by viral infections and a clearer pattern seems to be drawn by studies. It has been shown that the phylum Firmicutes (*Staphylococcus* and *Streptococcus *spp.) and Proteobacteria (*Haemophilus *spp., *Moraxella *spp*., Pseudomonas *spp*., Acinetobacter *spp*.*) are increasing in abundances in patients with influenza [212, 214–216]. However, those specific phyla are known to be seasonally fluctuant and Proteobacteria are dominant in the nasopharyngeal microbiome during fall–winter [217]. Few studies have been focusing on the lower airways, but it seems that the same pattern with an increase of Proteobacteria in the microbiome of the lung at least in patients with chronic lung diseases [218] and in vitro model [219, 220].

In patients infected with SARS-CoV-2, only a few studies have been performed on the impact on the lung microbiome. It seems that as for other respiratory viruses such as influenza the microbiome of the lung is affected during the infection and a shift toward a dominating Proteobacteria is observed. Shen et al*.* compared 8 patients with COVID-19 to 25 patients with community-acquired pneumonia (CAP), and 20 healthy controls [221]. They observed that the microbiome of the patient with COVID-19 was more closely related to the one of the CAP patients, the microbial composition in the bronchoalveolar lavage fluid (BALF) was dominated by either pathogenic bacterial strains or commensal bacteria commonly found in the upper respiratory tract. A second study with a slightly bigger cohort of 20 patients also showed that the microbiome of the lung shift to a mono-specific microbiota dominated by a Proteobacteria, which in this study was mostly *Acinetobacter* [222]. The analysis of the fungome also showed that *Cryptococcus* was the most dominant fungi which might be the results of an immune defect. A study based on a bigger cohort, comparing 62 COVID-19 patients to 125 non-COVID-19 pneumonia showed that COVID-19 patients had a reduced alpha diversity and the presence of potentially pathogenic microbes was detected in 47% of the cases [223]. Those studies do not imply causality or kinetic of infections, but it seems most likely that the viral infection by SARS-CoV-2 induces a dysbiosis of the lung which might, in turn, cause a secondary bacterial or fungal infection increasing the severity and fatality of the disease.

#### Gut microbiome

Although the respiratory organs is the main target for the clinical presentation of SARS-CoV-2 infection, gastro-intestinal symptoms have been reported on a subset of patients [224, 225]. Indeed ACE2, the receptor for SARS-CoV-2 spike protein is highly expressed in intestinal enterocytes [226, 227]. Moreover, viral RNA can sometimes be detected in rectal swabs after nasopharyngeal swabs have tested negative, suggesting the importance of the gut as a secondary niche for SARS-CoV-2 [228, 229]. In an in vitro intestinal organoid infection model, Lamers et al. demonstrated that enterocytes can be infected by SARS-CoV-2 and more importantly, the intestine may serve as an additional niche for SARS-CoV-2 to replicate within the human body and imply a secondary faecal-oral transmission route besides aerosols [230, 231].

Several symptoms of COVID-19 are directly implicating gut function such as digestive symptoms, vomiting and diarrhea [7, 8]. Gastro-intestinal symptoms are often link to severe COVID-19 complications [232]. The presence of digestive symptoms such as diarrhea, are known to be associated with a dysbiosis of the gut microbiome [233]. Furthermore, the role of the gut microbiome in the predisposition to and severity of viral infection is already recognized [234, 235]. This role is mostly due to the impact of the gut microbiome on the systemic immune system. The healthy gut microbiome produces bacterial metabolites which help to maintain the intact epithelial integrity, regulatory T-cell development, and low inflammatory immune state [236]. The major class of metabolites are short-chain fatty acids (SCFAs) such as acetate, propionate, and butyrate which promote the development of regulatory T cells [237, 238], induce “tolerogenic” immune response [239] and limit autoimmunity [240, 241]. On the other hand, SCFAs also promote immune response to pathogens via the production of AMPs and defensins [242], IL-18 and LL-37 production [243, 244]. A dysbiosis of the gut microbiome and especially in the microbes involved in the production of such metabolites will favor infection.

Several studies have been performed to evaluate the impact of COVID-19 on the gut microbiome. The first study on 15 patients demonstrated a gut microbiome fingerprint characterized by a decrease of the beneficial commensals and increase of opportunistic pathogen [245]. This dysbiosis was associated with the severity of the disease. The abundance of *Coprobacillus*, *Clostridium ramosum*, and *Clostridium hathewayi* correlated positively with COVID-19 severity while the abundance of *Faecalibacterium prausnitzii* showed the opposite effect. The dysbiosis was prolonged through the time of hospitalization despite the clearance of SARS-CoV-2 and resolution of the respiratory symptoms. Furthermore, the dysbiosis was also associated with higher levels of the virus in the feces. The species *Bacteroides dorei*, *Bacteroides thetaiotaomicron*, *Bacteroides massiliensis*, and *Bacteroides ovatus* correlates negatively with the SARS-CoV-2 load in fecal samples which would be explained by the fact that that those species downregulate the expression of ACE2 in the murine gut.

A study on 30 patients with COVID-19 also showed that the gut microbiome was associated with a dysbiosis compared to healthy controls. COVID-19 patients showed a significantly reduced bacterial diversity due to an overgrowth of opportunistic pathogens (*Streptococcus*, *Rothia*, *Veillonella* and *Actinomyces*) and a decrease of beneficial symbionts [246]. Another group worked on the transcriptional activity of SARS-CoV-2 in the feces of 15 patients with COVID-19 and showed that the gut microbiome was correlated to the viral transcriptional activity [247]. The fecal microbiome characterized by higher abundances of bacterial species *Collinsella aerofaciens, Collinsella tanakaei, Streptococcus infantis,* and *Morganella morganii* were typical of a high SARS-CoV-2 infectivity. On the other hand, fecal samples with low-to-no SARS-CoV-2 infectivity showed a higher abundance of *Parabacteroides merdae, Bacteroides stercoris, Alistipes onderdonkii,* and *Lachnospiraceae bacterium,* which are known producer of SCFA.

Finally, a study performed in 31 patients with COVID-19 showed that a blood proteomic risk score based on 20 proteomic biomarkers was associated with the severity of COVID-19 [248]. The authors of this study also correlated the gut microbiome to the PRS and found that 20 most important OTUs belonging to the *Bacteroides*, *Streptococcus* and *Lactobacillus* genus, Ruminococcaceae and Lachnospiraceae family and Clostridiales order. Those 20 OTUs could predict the PRS with good power (*R*^2^ = 0.59) especially in comparison to classical demographic and lab parameter such as age, BMI, sex, blood pressure and blood lipids. Those results indicate that the change in the microbiome might precede the changes in the blood proteomic biomarkers. Furthermore, this study showed a negative correlation between the *Bacteroides* genus, *Streptococcus* genus and Clostridiales order with most of the pro-inflammatory cytokines, while *Ruminococcus* and *Lactobacillus* genus were positively correlated.

The gut homeostasis has also been linked to ACE2 which regulates via a renin-angiotensin system (RAS) independent function the amino acid homeostasis and expression of antimicrobial peptides. ACE2-KO mice showed a decreased expression of antimicrobial peptides and exhibited altered intestinal microbial composition [249]. Furthermore, other coronavirus cause a downregulation of ACE2 levels in tissues to improve the viral replication efficiency and pathogenicity and it has been hypothesized than SARS-CoV-2 might act the same way [250, 251]. The link between COVID-19 and gut dysbiosis can be explained by the ACE2 imbalance, which bring together the development of the virus in the gut and the viral load and the gut dysbiosis.

#### Gut–lung axis

As both lung and gut microbiome seems imbalanced during COVID-19, it highlights once more the systemic impact of the immune response and the link between the gut and the lung. This link called the “gut–lung axis” conceptualize that the gut microbiome composition influences the lung’s immune response and by extension the lung microbiome and infection in the lung can also switch the gut microbiome composition toward pro-inflammatory status [252–254]. This concept introduces the impact of host–microbe as well as microbe–microbe interactions on localized and systemic immune response and the course of respiratory diseases. The gut is primordial to prime the mucosal immune response, and a perturbation of the normal gut microbiota may be associated with the development of an abnormal systemic mucosal response meaning the mucosal immunity is disturbed at distal mucosal sites, including the lung [255, 256]. This can be mediated by the migration of immune cells from the gut to the different organs through the lymphatic system [257]. Activated T and B cells can also move into the circulation and migrate from intestinal to the bronchial epithelium and lymphoid tissues [258]. While the immune system regulates the translocation of bacterial cells in the bloodstream, surviving bacteria, fragments of dead bacteria, bacterial metabolites such as SCFAs travel through the systemic circulation [256, 259]. Those factors will modulate distally the lung immune response [254, 260, 261]. The gut–lung axis and the microbiome of both niches are of high importance in many respiratory diseases and especially in ARDS, which is a common and severe complication of COVID-19 [262, 263]. Therefore, it seems that lung microbiome and gut microbiome are good biomarkers to predict the predisposition to COVID-19 and the severity of the outcomes. Furthermore, discussion and clinical trials on the use of microbiome transplantation and probiotics in COVID-19 patients to reduce the severity and infection are ongoing [264–266].

#### Microbiome–viral interaction

The lung and gut microbiome of healthy person present some specific composition such as a lung microbiota is dominated at the phylum level by *Firmicutes*, *Bacteroidetes*, *Proteobacteria*, *Fusobacteria* and *Actinobacteria* and at the genus level, Streptococcus and the anaerobic genus *Prevotella* and *Veillonella* originating from the upper airways are the most predominant [157–159]. In the gut, the homeostasis of the microbiota and low abundance of proteobacteria is crucial as it has been linked with less risk of obesity, cardiac disease and infection [267, 268]. The microbiome is highly personalized, and many lifestyle factors are influencing the microbiome. Those changes impacted mostly the diversity of the microbiome and therefore the protective role which relies on the competition between microorganisms and functional redundancy. By occupying the niche, the commensals microbes avoid the growth of potentially harmful pathogens [171]. Furthermore, a dysbiosis in the microbiome will triggers a change from commensal relationship to pathogenic relationship [269, 270]. In many cases, the decrease of diversity will increase the competition between the survivors and highly competitive opportunistic pathogens such as ESKAPE pathogens or Staphylococcus will become the dominant member of the microbiome.

A pathogen-dominated microbiome can also lead to an increase in co-infection. For several viral respiratory infections, the interaction between virus and commensal bacteria may result in a pathogenic synergism and complicate the course of diseases leading to increased morbidity and mortality [271, 272]. The mechanism behind the poor outcome of bacterial co-infection in underlying viral infection is complex, often pathogen-specific, and involves viral–bacterial interaction (direct) and their interaction with the host’s immunity (indirect) [273, 274]. One of the best-studied examples of viral–bacterial synergism is the interaction between influenza A virus and the bacterial pathogens, *S. aureus* and *S. pneumoniae* [275]. Although both bacterial pathogens may be a harmless commensal, in certain circumstances, these bacteria may undergo a transition from harmless commensals to invasive infectious agents [276]. Viral infection may facilitate the transition from carriage to infection. It is postulated that an initial viral infection may cause cellular damage to the respiratory epithelia, such as cell tight junctions, thus leading to a release of pro-inflammatory and danger signals [277]. This damage may expose the basal membrane and other important attachment sites for bacterial adhesion, hence leading to increased adherence and expansion of opportunistic pathogens [277, 278]. The adhesion of bacterial cells to cellular structures and epithelia is the first and essential step to enable entry and to initiate bacterial infection [274, 279]. In vitro and in vivo experimental data suggest that underlying influenza A infection can enhance *S. aureus* adhesion to the cell surface [280, 281]. In a murine model, the clearance of *S. aureus* was impaired by a concomitant influenza infection, hence increasing their susceptibility to *S. aureus* infections [282]. Of course, the virus-induced cellular damage is not the only factor for cellular damage, which can promote bacterial adhesion and invasion. Many predisposing factors, pre-existing conditions and underlying diseases may equally influence the propensity to acquire bacterial-viral co-infection.

Direct viral-bacterial interaction has also been demonstrated in numerous in vitro and animal studies. For example, *S. aureus* colonization was shown to affect viral load and influenza virus clearance in animal models [283–285]. Whereas virus-virus interactions are mostly competitive in nature [286], bacteria-virus interactions have been described as synergistic. This implies that potential virulence may be enhanced due to the virus-bacteria interaction [286]. One explanation could be that the shift in the local microbiota-equilibrium, promote commensal bacterial-bacterial interaction in the battle for space and nutrients [287]. Another factor, which should be considered is the immune phenotype associated with the composition of the commensal bacterial population. As an example, certain host immune phenotype is associated with the colonization with *S. aureus* [288–291]. As a result of a viral infection, type I interferon (IFN) production may be induced and interferes with the Th1 and Th17 immune response, which is essential for efficient clearance of *S. aureus* [292, 293]. Type I IFN inhibits the IL-23-dependent induction of Th17 immunity in the respiratory tract and consequently leading to lower levels of IL-17 producing CD4+ and γδ T cells and ultimately less IL-17 and IL-22 production. Both cytokines are important for *S. aureus* carriage [289]; IL-17 is essential for the clearance of *S. aureus* [292], whereas IL-22 regulates the antimicrobial-peptide, such as defensins, production by the innate immune cells [293]. Consequently, it is possible that a viral infection can modify the local immune phenotype, which may drive a commensal population into a dominating pathogen through dysbiosis, thus causing secondary infections. Due to the lack of high-quality experimental data on the viral–bacterial interaction for SARS-CoV-2 and *S. aureus* or other commensals, it is not possible to postulate to which extent these factors apply to the occurrence of bacterial co-infections and severity of SARS-CoV-2 infections. Further experimental studies on the viral–bacterial interaction of SARS-CoV-2 with important facultative pathogenic commensal bacteria are needed to elucidate the occurrence of bacterial co-infections in COVID-19 patients.

### Influence of co-infection on the course of the disease

Dysbiosis and immune imbalance will lead to microbial infection. Infections with more than one pathogenic agent may overwhelm the immune system, and the outcome is more or less unpredictable. At the time of publication, there is still limited data on the outcome and clinical presentation of multi-pathogen infection involving SARS-CoV-2. Nevertheless, it has been reported that secondary co-infections in a primary viral infection can lead to complications and negative affect the course of infection [271, 272].

#### Viral co-infections in COVID-19 patients

There are several published reports on co-infections of hospitalized COVID-19 patients with other respiratory pathogens, with influenza A and respiratory syncytial virus (RSV) being the most common co-infecting viral pathogens identified [294, 295]. Available data on the co-infections are limited mostly to case reports, and therefore, it is challenging to draw any conclusions from published data [294, 296]. Since most of these publications only report simultaneous detection of viral nucleic acid, it is not possible to assess, if certain viral respiratory infections subsequentially facilitate SARS-CoV-2 infections. Although the exact effect and outcome of patients infected with both influenza A and SARS-CoV-2 is still unclear, pre-publication data from a preprint on *medRxiv* suggest that co-infection may have a significant impact on morbidity and mortality, compared to influenza A or COVID-19 alone [297]. The odds of ventilator use, ICU admission and death are much greater in combined infections than in independent infections. Interestingly, the data suggested that the risk of testing positive for SARS-CoV-2 was 68% lower among influenza cases, suggesting potential pathogen interaction and competition. The first wave of the COVID-19 pandemic hit the northern hemisphere in the latter part of the flu season. Therefore, the number of cases of influenza A-SARS-CoV-2 co-infections may not accurately represent the situation during the flu season.

In dengue endemic area, symptoms of COVID-19 can be mistaken for dengue fever as they are difficult to distinguish [298–300]. It has been reported that there is a potential cross-reactivity between SARS-CoV-2 and dengue viruses, which may have an effect on the accuracy of rapid serologic testing [301]. A similarity of the protein structure in an in-silico analysis between the SARS-CoV-2 spike protein and dengue envelope protein has been suggested as a potential mechanism for this cross-reactivity [302]. Most available data of dengue and SARS-CoV-2 co-infection are limited to case reports. Nonetheless, a cohort study from Argentina suggested that co-infections with both viruses does not worsen the outcome of either SARS-CoV-2 or dengue infection alone [303].

#### Bacterial co-infections in COVID-19 patients

In prior influenza pandemics, disease severity and increased mortality have been linked with bacterial and fungal co-infection [275, 304, 305]. Therefore, there is a major concern, whether bacterial and fungal co-infection may influence the clinical presentation of SARS-CoV-2 infections in a similar manner to influenza. In contrast to the influenza virus, in previous coronavirus epidemics, such as SARS and MERS, very little evidence of bacterial and fungal co-infections was reported [306]. From the clinical point of view, this is a very important aspect for the optimal clinical management of COVID-19 patients, whether supportive antibiotics therapy may be beneficial in treating severe COVID-19 presentations.

During the early phase of the COVID-19 pandemic, published literature reported a low rate of bacterial co-infection in hospitalized COVID-19. However, there was a high use of broad-spectrum antimicrobial substances to anticipate secondary and co-infection in the early phase of the pandemic [295, 306, 307]. High-quality evidence is lacking but is desperately needed for antibiotic stewardship and clinical management of COVID-19 patients, bearing in mind that antimicrobial resistance was and still is a major health concern regardless of the pandemic [308]. Moreover, community-acquired co- and super-infection should be clearly distinguished from hospital-acquired infections due to the different predisposing factors for the acquisition of secondary pneumonia.

Recently, Garcia-Vidal and colleagues reported that only 3% (31/989) of COVID-19 patients admitted to a hospital in Barcelona, Spain presented with community-acquired bacterial infections. In these patients, *Streptococcus pneumoniae* and *Staphylococcus aureus* pneumonia were predominant [309]. Meanwhile, hospital-acquired infections were reported in 4% (43/989) of the patients, with well over half of these infections occurring in the critical care setting. As expected, *Pseudomonas aeruginosa*, *Escherichia coli*, *Klebsiella *spp and *S. aureus* were among the most common pathogen identified in patients with hospital-associated pneumonia (incl. ventilator-associated pneumonia) [309]. Similar observations have been reported by Hughes and colleagues, who reported a similarly low (6%; 51/836) occurrence of bacterial co-infections in patients admitted to a UK hospital [310]. In contrast, another study by Contour and colleagues reported a higher rate (28%; 26/92) of bacterial co-infection in COVID-19 patients admitted to the ICU of a French hospital, again with *S. aureus*, *Haemophilus influenzae*, *S. pneumoniae*, *Enterobacterales* and *P. aeruginosa* as the primary pathogens [311]. In this study, the patients presented with severe SARS-CoV-2 pneumoniae and, therefore, no direct comparison could be made with the aforementioned studies of Garcia-Visal et al*.* and Hughes et al*.* [309, 310]. In all these studies, the spectrum of the bacterial pathogen detected was similar. Commensal bacteria with pathogenic potential, such as *S. aureus*, *S. pneumoniae* and *H. influenzae* were commonly detected in COVID-19 patients, similar to the bacterial spectrum found in influenza patients [312]. The only difference is that the rate of bacterial co-infection in influenza is much higher than in COVID-19 patients, which may be an indication of co-incidental finding [313–315]. A meta-analysis by Klein et al. reported bacterial co-infection rates for influenza ranging from 2 to 65%, with *S. pneumoniae* and *S. aureus* as the most common co-infecting species [312]. Others reports on bacterial co-infections have been published [316, 317], but were not discussed in detail in this review due to the small sample size and a case-report nature of the data presented.

The comparability of data on co-infections in COVID-19 patients relies on several essential aspects. There is no consensus definition for co-infection and secondary infection. Microbial sampling techniques and detection techniques are variable. Moreover, the distinction between contamination and true infections for several microorganisms, such as *Candida *spp, *Enterococcus *spp and coagulase-negative staphylococci, is not always clear cut. Despite some supporting evidence of the importance of bacterial co-infection in viral respiratory diseases, this aspect is still understudied in the context of COVID-19. Indeed, the diagnosis of bacterial co-infection in COVID-19 patients is a challenge. These bacteria may be part of the commensal bacteria, associated with an underlying chronic disease or acquired during hospitalization. While antibiotics are ineffective to treat SARS-CoV-2 infection, they are prescribed frequently for COVID-19. This further complicates the detection of bacterial co-infection due to the reduced sensitivity of conventional culture-based detection methods. Since there is no consensus reporting standard for studies and reports on bacterial co-infection for COVID-19, the quality of clinical data is heterogeneous. These aspects should be taken into consideration for future prospective studies.

As infection cases surge in tropical and sub-tropical regions, where tuberculosis (TB) is endemic, evidence on the potential interaction between TB and COVID-19 accumulate. In a meta-analysis of six studies from China with small study sample, TB prevalence was described to be higher among patients with severe COVID-19 compared to non-severe cases and that the risk of TB-related mortality was 1.4 times higher in COVID-19 patients [318]. Another study based on data from the Philippines reported an increased risk (2.17 times higher) of death in TB patients co-infected with COVID-19 [319]. However, the definitive effect of COVID-19 on TB progression/sequelae is still unclear. Furthermore, evidence on the causative association is still lacking. A global study initiative on TB and COVID-19 co-infections supported by the World Health Organization is currently underway [320].
